# Complete Genome Sequence of Duncaniella muris Strain B8, Isolated from the Feces of C57/BL6 Mice

**DOI:** 10.1128/MRA.00566-19

**Published:** 2019-07-25

**Authors:** Sou Miyake, Yichen Ding, Melissa Soh, Henning Seedorf

**Affiliations:** a Temasek Life Sciences Laboratory, Singapore; b Department of Biological Sciences, National University of Singapore, Singapore; Indiana University, Bloomington

## Abstract

Here, the complete genome sequence of Duncaniella muris strain B8 is presented. The anaerobic strain was isolated from the feces of C57/BL6 mice and is closely related to *D. muris* strain DSM 103720, which is the type strain of the recently proposed genus *Duncaniella* of the *Muribaculaceae*.

## ANNOUNCEMENT

The *Bacteroidetes* family *Muribaculaceae* has been proposed only recently (1), and few isolates and/or genome sequences are publicly available from members of this family. This regards specifically the *Muribaculaceae* genus Duncaniella (2), which has currently only one cultured representative, the D. muris type strain DSM 10372 (2, 3). *Duncaniella* spp., and specifically *D. muris*, are highly abundant in the murine intestinal tract, but limited genomic data are currently available for *Duncaniella* isolates (2). In this study, *D. muris* strain B8 was obtained from the feces of C57/BL6 mice (experimental procedures involving animals were performed according to the approved IACUC protocol, TLL-16–016) using an anaerobic cultivation medium described by Harris et al. (4). The 16S rRNA gene of strain B8 and the *D. muris* type strain share 99.9% sequence identity, but it can be hypothesized that differences between the two strains are detectable at the genome level, since they were cultivated at different laboratories, from mice with different genotypes, and under different experimental conditions.

Genomic DNA was prepared using two rounds of phenol-chloroform purification as previously described (5) with modifications. Ten micrograms of prepared and purified genomic DNA was used for library construction for PacBio RS II single-molecule sequencing according to the manufacturer’s instructions. PacBio sequencing generated 111,150 reads with a total of 1,244,160,060 bases. Raw PacBio sequencing reads were corrected, trimmed, and *de novo* assembled into a single contig using default settings in Canu version 1.8 (6). The assembled genome was annotated with the Rapid Annotation of microbial genomes using Subsystems Technology (RAST) server (7). RNAmmer (8) was used to annotate and verify the RNA genes. The contig had a total assembly size of 3,384,950 bp, with a G+C content of 50.90% and an average coverage of 365.9× across the genome. No plasmid was identified. The B8 genome encodes 3,034 protein-coding sequences (CDSs) and 72 RNAs (12 rRNA and 60 tRNA genes). The calculation of average nucleotide identity (ANI) between B8 and the draft genome of type strain DSM 103720 using the ANI calculator (9) suggested an ANI value of 98.02%, whereas prediction of *in situ* DNA-DNA hybridization (isDDH) on the CGDC version 2.0 Web service (10) suggested an isDDH value of 86.6% (using formula 2). In addition, the G+C content of strain B8 is nearly identical to that of the *D. muris* type strain DSM 103720 (50.8%). Considering that the proposed species boundary cutoff for ANI is 95 to 96% (9) and that for DDH is 70% (10), these data indicate that B8 is a strain of *D. muris*.

The genome presented here is currently the only complete *Duncaniella* genome. The high abundance of *Duncaniella* species in murine intestinal tracts (2, 11) and the host-specificity of the *Muribaculaceae* species (12) highlight the importance of obtaining more genomic information for members of this family. Strain B8 will therefore serve as an important resource for comparative analysis with other strains and may help to elucidate specific adaptations of *Muribaculaceae* strains to the intestinal tract.

### Data availability.

This whole-genome shotgun project has been deposited in GenBank under the accession no. CP040121. The version of the described assembly in this paper is the first version, CP040121.1. PacBio RS II raw data are available under the Sequence Read Archive accession no. SRX5827558.
